# Differences in the renal antifibrotic signaling of serelaxin and zaprinast

**DOI:** 10.1186/2050-6511-16-S1-A99

**Published:** 2015-09-02

**Authors:** Veronika Wetzl, Lothar Faerber, Franz Hofmann, Elisabeth Schinner, Jens Schlossmann

**Affiliations:** 1 Department of Pharmacology and Toxicology, Institute of Pharmacy, University of Regensburg, Germany; 2 Novartis Pharma GmbH, Nuremberg, Germany; 3 Carvas, Technical University Munich, Munich, Germany

## Background

Kidney fibrosis is frequently observed in cardiorenal diseases. Cyclic guanosine monophosphate (cGMP) serves as the most important second messenger of nitric oxide (NO) and has shown antifibrotic effects at enhanced levels in several experimental models of kidney diseases. Antifibrotic effects of cGMP signalling via cGMP-dependent protein kinases (cGK), in particular cGKI, have already been shown. Serelaxin and zaprinast are both able to increase cGMP signalling via influencing two independent pharmacological targets. Serelaxin is currently in phase III development for acute heart failure and has shown antifibrotic properties in several *in vitro* and *in vivo* experiments. It is discussed that serelaxin inhibits TGF-β signalling via RXFP1 receptor. Involvement of NO / cGMP in this process has also been revealed. Zaprinast is a selective inhibitor of phosphodiesterase V, which leads to enhanced levels of cGMP by blocking cGMP degradation.

## Purpose

The aim of the study was to compare renal antifibrotic effects of the cGMP enhancing serelaxin and the cGMP-degradation-limiting zaprinast in a model of interstitial kidney fibrosis. We hypothesize that antifibrotic properties of both compounds are mediated through cGMP dependent protein kinases, namely cGKI.

## Methods

Kidney fibrosis was induced by unilateral ureteral obstruction (UUO) in wild type (WT) and cGKI knock out (KO) mice. Serelaxin was administered through osmotic minipumps (0.5 mg/kg/d) immediately after UUO for seven days. Zaprinast was administered through intraperitoneal injections once a day (10 mg/kg/d) immediately after UUO for seven days. cGMP levels were measured via ELISA. Antifibrotic effects were assessed by biomarkers indicating myofibroblast differentiation, extracellular matrix (ECM) accumulation or degradation via mRNA- and protein expression.

## Results

The levels of cGMP were enhanced in kidney tissue after treatment with Serelaxin or Zaprinast. In WT, Serelaxin and Zaprinast significantly reduced renal interstitial fibrosis assessed by remodeling biomarkers, e.g. α-SMA, total collagen, Col1a1, fibronectin and gelatinases. In contrast to Zaprinast, Serelaxin showed no antifibrotic effects on mRNA and protein expression of ECM accumulation in cGKI-KO.

## Conclusion

These results indicate that Serelaxin and Zaprinast have comparable antifibrotic effects in the kidney of WT animals. The involvement of cGMP signalling through cGKI was shown in both treatment options, e.g. regarding myofibroblast differentiation. Due to reduced extracellular matrix in cGKI-KO after Zaprinast treatment, additional cGKI independent mechanisms are supposed for antifibrotic signalling of Zaprinast.

